# Consumption of red meat, genetic susceptibility, and risk of LADA and type 2 diabetes

**DOI:** 10.1007/s00394-020-02285-2

**Published:** 2020-05-22

**Authors:** Josefin E. Löfvenborg, Emma Ahlqvist, Lars Alfredsson, Tomas Andersson, Leif Groop, Tiinamaija Tuomi, Alicja Wolk, Sofia Carlsson

**Affiliations:** 1 Institute of Environmental Medicine, Karolinska Institutet, Box 210, 171 77 Stockholm, Sweden; 2 Department of Clinical Sciences, Lund University, Malmö, Sweden; 3 Center for Occupational and Environmental Medicine, Stockholm County Council, Stockholm, Sweden; 4 Institute for Molecular Medicine Finland (FIMM), University of Helsinki, Helsinki, Finland; 5 Division of Endocrinology, Abdominal Centre, Helsinki University Hospital, Helsinki, Finland; 6 Folkhälsan Research Center, Helsinki, Finland

**Keywords:** Latent autoimmune diabetes in adults, Red meat intake, HLA, *TCF7L2*, Family history, Interaction

## Abstract

**Purpose:**

Red meat consumption is positively associated with type 1 (T1D) and type 2 (T2D) diabetes. We investigated if red meat consumption increases the risk of latent autoimmune diabetes in adults (LADA) and T2D, and potential interaction with family history of diabetes (FHD), HLA and *TCF7L2* genotypes.

**Methods:**

Analyses were based on Swedish case–control data comprising incident cases of LADA (*n* = 465) and T2D (*n* = 1528) with matched, population-based controls (*n* = 1789; *n* = 1553 in genetic analyses). Multivariable-adjusted ORs in relation to self-reported processed and unprocessed red meat intake were estimated by conditional logistic regression models. Attributable proportion (AP) due to interaction was used to assess departure from additivity of effects.

**Results:**

Consumption of processed red meat was associated with increased risk of LADA (per one servings/day OR 1.27, 95% CI 1.07–1.52), whereas no association was observed for unprocessed red meat. For T2D, there was no association with red meat intake once BMI was taken into account. The combination of high (> 0.3 servings/day vs. less) processed red meat intake and high-risk HLA-*DQB1* and -*DRB1* genotypes yielded OR 8.05 (95% CI 4.86–13.34) for LADA, with indications of significant interaction (AP 0.53, 95% CI 0.32–0.73). Results were similar for the combination of FHD-T1D and processed red meat. No interaction between processed red meat intake and FHD-T2D or risk variants of *TCF7L2* was seen in relation to LADA or T2D.

**Conclusion:**

Consumption of processed but not unprocessed red meat may increase the risk of LADA, especially in individuals with FHD-T1D or high-risk HLA genotypes.

**Electronic supplementary material:**

The online version of this article (10.1007/s00394-020-02285-2) contains supplementary material, which is available to authorized users.

## Introduction

Consumption of red meat, especially processed meat, has been associated with an increased risk of type 2 diabetes (T2D) [1] as well as childhood type 1 diabetes (T1D) [2–4]. Several compounds in red meat, some of which are particularly abundant in processed meat products, may affect diabetes risk including advanced glycation endproducts (AGEs), sodium, iron, and nitrates, nitrites, and nitrosamines. Proposed mechanisms involve promotion of insulin resistance (e.g., inflammation and oxidative stress) as well as detrimental effects on pancreatic beta cells [5]. The effect may be stronger in genetically susceptible individuals. In support hereof, a study nested within the Health Professionals Follow-up Study reported interaction between red meat and a T2D genetic risk score (GRS) composed of ten single nucleotide polymorphisms (SNPs) in relation to the risk of T2D [6]. Moreover, the association with red meat consumption and islet autoimmunity and T1D has primarily been observed in children carrying high-risk human leukocyte antigen (HLA) genotypes [3, 7].

Latent autoimmune diabetes in adults (LADA) is a common, hybrid form of diabetes with a pathogenesis involving autoimmune destruction of pancreatic beta cells as well as insulin resistance [8]. Genetically, LADA resembles T1D with excess risk conferred by family history of diabetes (FHD) of T1D and HLA genotypes [9, 10]. Genetic similarities with T2D have also been seen including an association with transcription factor 7-like 2 (*TCF7L2)* [11] and FHD of T2D [9]. Red meat intake may affect the risk of LADA through the same mechanisms potentially linking it to risk of T1D or T2D and the association may depend on genetic factors, but this remains to be explored.

Our objective was to investigate the association between consumption of unprocessed and processed red meat and risk of LADA and T2D, and the potential interaction with FHD of T1D, T2D, and genotypes of HLA and *TCF7L2.* Furthermore, we aim to explore whether the associations may be mediated by effects of insulin resistance, beta cell function, or autoimmunity.

## Subjects and methods

### Study design and population

Analyses were based on data from the Swedish population-based case–control study ESTRID (Epidemiological Study of Risk Factors for LADA and Type 2 Diabetes; https://ki.se/imm/estrid; Supplementary Fig. 1) [12]. Incident cases of LADA and T2D are recruited from ANDIS (All New Diabetics in Scania); an extensive registry and biobank aiming to genetically and clinically characterize all new diabetes patients in Scania [13], and ANDiU (All New Diabetics in Uppsala); a sister study in Uppsala County. Since 2010, all incident cases of LADA and a random sample of T2D cases recorded in ANDIS and ANDiU (starting 2012; 5% of ESTRID cases) have been invited to participate by responding to a detailed health- and lifestyle questionnaire. Median time between diagnosis of LADA or T2D and questionnaire response is 6.2 months and 5.0 months, respectively. Population-based, diabetes-free controls are identified through the Swedish Population Register and matched to cases by time and region (incidence-density sampling [14]). Response rate is 80% among cases and 62% among controls. The ESTRID controls provide questionnaire data but no blood samples. For analyses of genetic risk factors, data for diabetes-free controls from the EIRA Study (Epidemiological Investigation on Rheumatoid Arthritis) is used [15]. These controls, from here on referred to as “genetic controls”, are randomly selected from the population and matched to the diabetes cases by age and sex. They answer a similar questionnaire as the cases and provide blood samples for genetic analyses.

Eligible for the present study were all cases and controls included in ESTRID between 2010 and July 2017 with complete covariate information and reported energy intakes within 3 standard deviations (SD) from the log_e_-transformed sex-specific mean. The analytic sample included 465 LADA cases, 1528 T2D cases, and 1789 controls. The genetic controls were collected 2005–2014, aged ≥ 35 years, and with complete covariate information including ≥ 1 of the genetic factors (HLA or/and *TCF7L2*) (*n* = 1553). The study was approved by the Regional Ethical Review Board in Stockholm and all participants provided informed consent.

### Classification of diabetes

Classification into diabetes subtype was based on age at diagnosis, glutamic acid decarboxylase autoantibodies (GADA), and fasting C-peptide. Details of the serological assay methods have been described previously [13]. GADA was determined using enzyme-linked immunosorbent assay (ELISA) with 84% sensitivity and 98% specificity at 10.7 IU/mL cut-off level [16]. Values > 250 IU/mL were censored at 250 IU/mL. C-peptide concentration was measured using Cobas e601 analyzer (Roche Diagnostics, Germany) or IMMULITE 2000 (Siemens Healthcare Diagnostics Product Ldt., UK). Cases of LADA had age ≥ 35 years, GADA ≥ 10 IU/mL, and C-peptide ≥ 0.2 nmol/L (IMMULITE) or ≥ 0.3 nmol/L (Cobas e 601). T2D was defined as age ≥ 35 years, GADA < 10 IU/mL, and C-peptide ≥ 0.60 nmol/L (IMMULITE) or ≥ 0.72 nmol/L (Cobas e 601). Homeostatic model assessment of insulin resistance (HOMA-IR) and beta cell function (HOMA-B) were calculated based on fasting levels of plasma glucose and C-peptide [17].

### Diet and other covariate assessment

Dietary intake was assessed using a 132-item semi-quantitative food frequency questionnaire (FFQ). Participants were asked to report how often, on average over the previous year, they had consumed various foods by indicating one of eight pre-defined frequency categories ranging from “0 times per month” to “≥ 3 times per day”. The patients were specifically instructed to report their habitual intake reflecting the year preceding diagnosis. Eleven questions concerned red meat consumption; five items on unprocessed red meat [pork, beef/veal, minced meat, offal (liver/kidney), and other meat], and six on processed red meat [sausages/hot dogs, Falun sausage, other sausages, cold cuts (ham/salami), bacon, black pudding, and liver paté]. Energy intake (kcal/day) was estimated based on age- and sex-specific serving sizes combined with nutrition values from the Swedish National Food Agency database. The FFQ has been validated for nutrients using fourteen 24-h recall interviews, with Spearman correlation coefficients of 0.65 for macronutrients and 0.62 for micronutrients [18]. The genetic controls answered identical questions on red meat consumption. As a whole, their FFQ was somewhat shorter with 124 items, hampering comparability of estimated energy intake.

Information on non-dietary covariates was derived from the questionnaire. Height and bodyweight were used to calculate BMI (kg/m^2^). Highest attained education level was categorized as primary school, upper secondary school, or university. Four pre-defined response options ranging from sedentary to regularly active were used to assess leisure-time physical activity during the preceding year (prior to diagnosis for patients). Smoking habits were categorized into never, former, or current smoker. Average daily alcohol intake was estimated from the FFQ and categorized into none, 0.01–4.9 g/day, 5–14.9 g/day, and ≥ 15 g/day. FHD of T1D (FHD-T1D) was defined as a first-degree relative with diabetes onset age < 40 years combined with insulin therapy, otherwise defined as FHD of T2D (FHD-T2D). When used in confounder adjustment in the main analyses, FHD was dichotomized into none or ≥ 1 relative with diabetes. The corresponding information was available for the genetic controls, with the exceptions of energy intake and FHD.

### Genotyping

Genotyping of patients was based on blood samples analyzed at the Clinical Research Center in Malmö, Sweden, using iPlex Gold Technology (Sequenom, San Diego, CA, USA). For a subset, missing genotypes were imputed using Infinium CoreExome v1.1 (Illumina, USA), based on the Haplotype Reference Consortium (https://www.haplotype-reference-consortium.org/; version r1.1 2016) reference panel. For genetic controls, genotyping was based on GWAS data from Illumina Global Screening array or Infinium Illumina 300K immunochip custom array (Illumina, USA). Based on three SNPs within the HLA region (rs3104413, rs2854275, rs9273363), participants were genotyped according to previously described methodology [19]. Based on the literature [20, 21] and frequency distributions in our study population, participants were categorized as carriers of high-risk HLA genotypes (*DR4-DQ8; DR4/3-DQ8; DR3/4; DR3/3; DR4/4,* and DRB1*0301-DQA1*0501-DQB1*0201) or low-/intermediate-risk genotypes (*DR3/x; DR4/x; DR4-DQ7,* and *DRx/x*, where *x* = neither *DR4* nor *DR3*). For *TCF7L2* rs7903146, participants were classified as risk genotype carriers if they had at ≥ 1 risk allele (i.e., TT or TC).

### Statistical analysis

Characteristics for patients and controls were presented as proportions, means, or medians (skewed data), with SD (means) or interquartile range (IQR; medians). Characteristics of study participants are also presented by quartiles of consumption of unprocessed red meat and processed red meat in supplementary tables. Chi-square test (proportions), Student’s *t* test (means), and Kruskal–Wallis *H* (medians) tests were used to calculate two-tailed *p* values.

Conditional logistic regression models were used to estimate odds ratios (OR) and 95% confidence intervals (CI) of LADA and T2D in relation to genotypes, FHD, and consumption of unprocessed and processed red meat. ESTRID controls were used in all analyses except those including genetic covariates. Model 1 was adjusted for age and sex. In model 2, additional adjustment was made for education, physical activity, smoking, alcohol, energy intake (continuous), and FHD. Model 3 additionally included BMI (continuous), a potential mediator. With regard to red meat consumption, results from model 3 is discussed in the text unless otherwise specified. Model 1 adjustment was used for the association between genotypes and diabetes (corresponding to Supplementary Fig. 2), and the analysis of LADA and T2D in relation to FHD (≥ 1 first degree relative) was adjusted according to model 3 except energy intake but with mutual adjustment for FHD-T2D and FHD-T1D. All covariates included were chosen based on previous knowledge on our study population and the literature. Red meat consumption was analyzed in quartiles (based on distribution among controls) and continuously per one daily serving increment [median serving size was 107.4 g (interquartile range, IQR 55.9) for unprocessed red meat and 46.3 g (IQR 26.1) for processed red meat]. The lowest consumption category was used as reference. Restricted cubic splines (model 3) with three knots were used to explore potential nonlinear relationships. Linear regression models (model 3) were used to explore the change in GADA (Tobit regression to account for the right-censoring at 250 IU/mL), HOMA-IR, and HOMA-B (log_e_-transformed) per one daily serving increment in red meat intake.

Attributable proportion (AP) due to interaction was estimated to examine the presence of interaction, defined as departure from additivity of effects, between processed red meat consumption [low (lowest quartile; 0–0.3 servings/day) or high (upper three quartiles)] and genotypes of HLA and *TCF7L2* as well as FHD-T1D and FHD-T2D on the risk of LADA and T2D. These analyses were adjusted according to model 3, except that genetic analyses did not include energy intake and FHD. AP > 0 with a 95% CI not including 0 indicate significant positive interaction [22].

Sensitivity analyses (model 3) were performed by additional adjustments for diet (fruits, vegetables, fatty fish, snacks, coffee, sweetened beverages) and mutual adjustment for unprocessed and processed meat. We also made restrictions to patients responding to the questionnaire within 6 months of diagnosis, currently on diabetes treatment that includes ‘diet modification’, and not reporting ‘major lifestyle changes’ after diagnosis of diabetes.

Statistical Analysis Software 9.4 (SAS Institute, USA) and Stata Statistical Software 14.2 (StataCorp, USA) (for spline modeling) were used for statistical analyses.

## Results

### Characteristics

In comparison with T2D, patients with LADA were less overweight and obese, less insulin resistant, but had worse beta cell function (Table 1). The proportion of high-risk HLA genotype carriers was equal in individuals with T2D and controls, but considerably higher in LADA. Individuals in the highest quartiles of red meat intake also had higher energy intake compared to those in the lowest quartiles (Supplementary Table 1). Among those in the highest quartile of unprocessed red meat intake, 40.7% were also in the highest quartile of processed red meat intake (not shown in table).

**Table 1:** Characteristics of ESTRID cases and controls

	Controls	Genetic controls	LADA	Type 2 diabetes	*p*^a^	*p*^b^
*n*	1789	1553	465	1528	–	–
Age, years, mean (SD)	58.6 (13.6)	57.6 (9.8)	59.1 (12.2)	63.2 (10.2)	< 0.0001	0.4400
Sex, % women	51.9	73.5	46.7	39.1	0.0035	0.0454
BMI, kg/m^2^, mean (SD)	25.9 (4.2)	25.4 (4.1)	28.2 (5.5)	31.1 (5.4)	< 0.0001	< 0.0001
Overweight (BMI ≥ 25), %	54.1	47.0	70.3	92.5	< 0.0001	< 0.0001
Obese (BMI ≥ 30), %	15.0	12.4	32.5	51.3	< 0.0001	< 0.0001
Family history of diabetes, % yes	24.3	–	43.1	49.6	0.0128	< 0.0001
Insulin treatment, %	–	–	42.4	5.8	< 0.0001	–
HOMA-IR, median (IQR)	–	–	2.8 (1.8–4.4)	3.6 (2.7–4.8)	< 0.0001	–
HOMA-B, median (IQR)	–	–	37.9 (14.4–68.3)	68.8 (43.5–94.0)	< 0.0001	–
GADA, IU/mL, median (IQR)	–	–	250 (29–250)	–	–	–
High-risk HLA genotype, %	–	31.8	61.2	31.5	< 0.0001	< 0.0001
TT/TC in *TCF7L2* rs7903146, %	–	46.3	52.1	52.5	0.8832	0.0423

^a^For comparison between LADA and type 2 diabetes

^b^For comparison between LADA and the internal ESTRID controls, except for the genotypes, where comparison is made between LADA and genetic controls

### Red meat intake, LADA and T2D

**Fig. 1:**
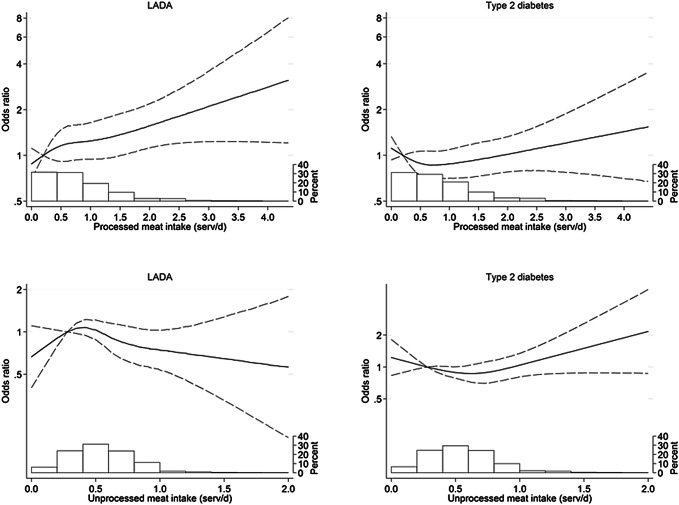
Restricted cubic spline models estimating ORs (solid line) with 95% CIs (dashed line) of LADA and type 2 diabetes in relation to consumption of unprocessed red meat and processed red meat, adjusted according to Model 3. The distribution of red meat intake in the study population is presented in the histogram at the bottom of each panel

Processed red meat consumption was positively associated with LADA; each additional daily serving was associated with OR 1.27 (95% CI 1.07–1.52) and an OR of 1.53 (1.08–2.16) was observed in the highest vs. lowest quartile. No association was seen with unprocessed red meat (OR per daily serving 0.69, 95% CI 0.46–1.03). For LADA, restricted cubic spline analysis indicated an increased risk that appeared to be linear for processed red meat consumption exceeding 1.5 servings/day, but no indication of an association with unprocessed red meat (Fig. 1).

T2D was not associated with processed or unprocessed red meat consumption after adjustment for BMI (Table 2, Fig. 1).

**Table 2:** ORs with 95% CI of LADA and type 2 diabetes in relation to intakes of unprocessed red meat and processed red meat, respectively

Type of red meat	Quartile (median, range, [serv/day])	LADA				Type 2 diabetes			
		Case/ control	OR (95% CI)^a^	OR (95% CI)^b^	OR (95% CI)^c^	Case/ control	OR (95% CI)^a^	OR (95% CI)^b^	OR (95% CI)^c^
Unprocessed red meat	Q1 (*m* = 0.2, 0–0.3)	106/434	1.00 (ref)	1.00 (ref)	1.00 (ref)	408/434	1.00 (ref)	1.00 (ref)	1.00 (ref)
	Q2 (*m* = 0.4, 0.3–0.5)	135/459	1.20 (0.90–1.60)	1.14 (0.84–1.53)	1.12 (0.82–1.52)	363/459	0.92 (0.75–1.13)	0.90 (0.72–1.13)	0.88 (0.69–1.13)
	Q3 (*m* = 0.6, 0.5–0.7)	115/435	1.10 (0.81–1.48)	1.05 (0.77–1.45)	1.02 (0.74–1.41)	358/435	0.95 (0.77–1.17)	0.98 (0.78–1.23)	0.85 (0.66–1.09)
	Q4 (*m* = 0.9, 0.7–4.1)	109/461	0.98 (0.72–1.32)	0.88 (0.63–1.22)	0.83 (0.59–1.16)	399/461	1.03 (0.84–1.27)	1.08 (0.86–1.36)	0.91 (0.70–1.18)
	Per 1 serv/day increment	*465/1789*	*0.92 (0.65–1.31)*	*0.78 (0.53–1.15)*	*0.69 (0.46–1.03)*	*1528/1789*	*1.24 (0.99–1.54)*	*1.31 (1.02–1.70)*	*1.07 (0.80–1.43)*
Processed red meat	Q1 (*m* = 0.2, 0–0.3)	84/436	1.00 (ref)	1.00 (ref)	1.00 (ref)	332/436	1.00 (ref)	1.00 (ref)	1.00 (ref)
	Q2 (*m* = 0.5, 0.3–0.7)	116/460	1.30 (0.95–1.78)	1.35 (0.98–1.87)	1.31 (0.94–1.82)	329/460	0.91 (0.74–1.13)	0.94 (0.74–1.18)	0.80 (0.62–1.04)
	Q3 (*m* = 0.8, 0.7–1.1)	117/449	1.33 (0.97–1.82)	1.36 (0.97–1.89)	1.28 (0.91–1.79)	374/449	1.01 (0.82–1.25)	1.01 (0.80–1.27)	0.80 (0.62–1.05)
	Q4 (*m* = 1.4, 1.1–4.6)	148/444	1.70 (1.25–2.31)	1.65 (1.18–2.31)	1.53 (1.08–2.16)	493/444	1.31 (1.06–1.60)	1.27 (1.01–1.61)	0.94 (0.72–1.22)
	Per 1 serv/day increment	*465/1789*	*1.35 (1.16–1.58)*	*1.32 (1.11–1.56)*	*1.27 (1.07–1.52)*	*1528/1789*	*1.22 (1.09–1.37)*	*1.19 (1.04–1.35)*	*1.06 (0.92–1.22)*

^a^Model 1 adjusted for age and sex

^b^Model 2 adjusted for age and sex + education, physical activity, smoking, alcohol intake, energy intake, and family history of diabetes

^c^Model 3 adjusted for age, sex, education, physical activity, smoking, alcohol intake, energy intake, family history of diabetes + BMI

### Genotypes, FHD, red meat, LADA and T2D

**Fig. 2:**
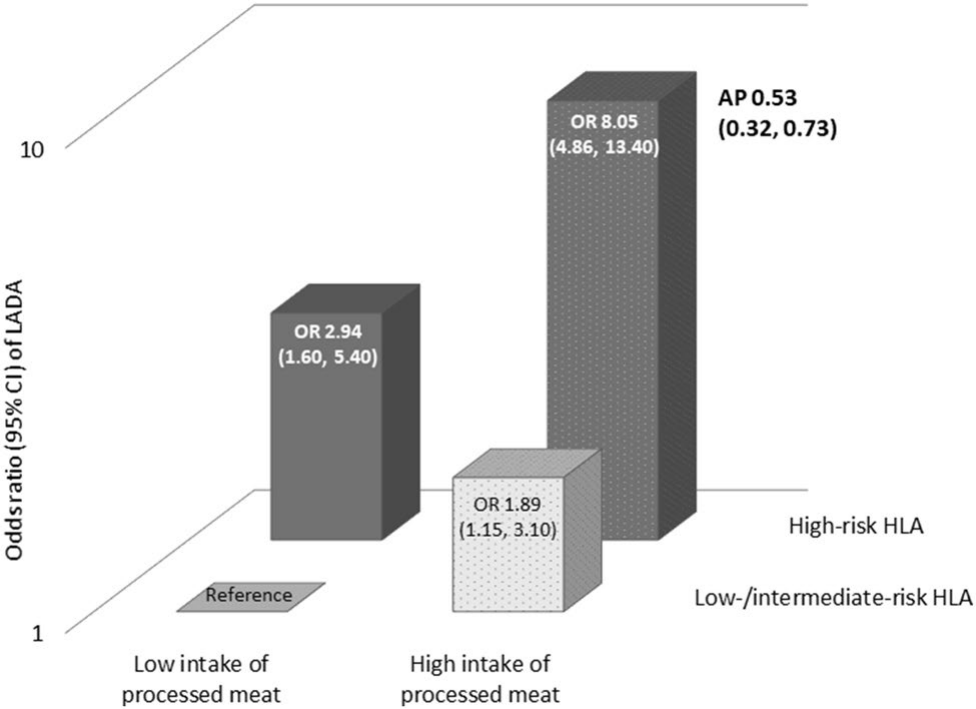
OR with 95% CI of LADA in relation to mutual exclusive combinations of processed red meat intake and HLA genotype. Adjustments were made for age, sex, education, physical activity, smoking status, alcohol consumption, and BMI

As shown previously in this population [9, 23], LADA was strongly associated with HLA genotypes and to a lesser extent also with genotypes of *TCF7L2* (Supplementary Fig. 2). Similarly, LADA was more strongly associated with FHD-T1D than with FHD-T2D. The combination of high-risk HLA genotypes and high consumption of processed red meat conferred an OR of 8.05 (95% CI 4.46–13.34) for LADA with AP estimated at 0.53 (95% CI 0.32–0.73) (Fig. 2). High consumption of processed red meat combined with FHD-T1D indicated similar results (OR 6.61, 95% CI 3.79–11.51; with AP 0.44, 95% CI − 0.14 to 1.02) (Table 3). There was no evidence of interaction between processed red meat intake and *TCF7L2* genotype or FHD-T2D in relation to LADA.

**Table 3:** OR with 95% CI of LADA and type 2 diabetes in relation to mutually exclusive combinations of processed red meat intake and genotype or family history of diabetes in first degree relatives

Genotype/family history	Consumption of processed meat	LADA				Type 2 diabetes			
		Cases	Controls	OR (95% CI)	AP (95% CI)	Cases	Controls	OR (95% CI)	AP (95% CI)
HLA genotype^a^									
Low-/intermediate-risk	Low	28	188	1.00 (reference)		182	188	1.00 (reference)	
Low-/intermediate-risk	High	121	414	1.89 (1.15; 3.10)		662	414	1.27 (0.92; 1.76)	
High-risk	Low	38	94	2.94 (1.30; 5.40)		79	94	0.92 (0.56; 1.51)	
High-risk	High	197	186	8.05 (4.86; 13.34)	0.53 (0.32; 0.73)	309	186	1.47 (1.02; 2.14)	0.19 (− 0.21; 0.59)
Family history of T1D^b^									
No	Low	76	427	1.00 (reference)		321	427	1.00 (reference)	
No	High	341	1319	1.31 (0.97; 1.76)		1135	1319	0.85 (0.68; 1.05)	
Yes	Low	8	9	3.40 (1.22; 9.43)		11	9	1.75 (0.63; 4.87)	
Yes	High	40	34	6.61 (3.79; 11.51)	0.44 (− 0.14; 1.00)	61	34	1.44 (0.84; 2.48)	− 0.11 (− 0.45; 1.00)
*TCF7L2* genotype^a^									
CC	Low	31	270	1.00 (reference)		121	270	1.00 (reference)	
CC	High	153	557	2.24 (1.45; 3.48)		465	557	1.47 (1.06; 2.03)	
TT/TC	Low	36	236	1.37 (0.80; 2.37)		141	236	1.44 (0.97; 2.14)	
TT/TC	High	164	477	2.82 (1.81; 4.37)	0.07 (− 0.27; 0.41)	507	477	2.09 (1.50; 2.89)	0.09 (− 0.22; 0.39)
Family history of T2D^b^									
No	Low	52	333	1.00 (reference)		169	333	1.00 (reference)	
No	High	248	1050	1.33 (0.94; 1.87)		634	1050	0.83 (0.63; 1.09)	
Yes	Low	32	103	1.73 (1.04; 2.88)		163	103	2.87 (1.97; 4.20)	
Yes	High	133	303	2.40 (1.64; 3.51)	0.14 (− 0.24; 0.52)	562	303	2.57 (1.92; 3.43)	− 0.05 (− 0.45; 0.34)

^a^Model adjusted for age and sex, education, physical activity, smoking, alcohol intake, and BMI

^b^Model adjusted for age and sex, education, physical activity, smoking, alcohol intake, energy intake, and BMI

T2D was associated with *TCF7L2* risk genotypes and FHD-T2D, but not with high-risk HLA genotypes and FHD-T1D (Supplementary Fig. 2). However, no evidence of interaction was found between intake of red meat and genotypes of *TCF7L2* or FHD-T2D on the risk of T2D (Table 3).

### Red meat intake, HOMA, and GADA

In T2D individuals, one additional daily serving of processed red meat was inversely associated with HOMA-B (− 6.7%, *p* = 0.0056) and positively associated with HOMA-IR (8.1%, *p* = 0.0029) (Supplementary Table 2). Unprocessed red meat intake was inversely associated with HOMA-B (− 12.2%, *p* = 0.0096) but not with HOMA-IR (Supplementary Table 2). In LADA patients, a decrease in HOMA-B and increase in HOMA-IR in relation to red meat intake, particularly processed red meat, was suggested but not significantly so (Supplementary Table 2). No associations were observed between red meat intake and GADA.

### Sensitivity analysis

Additional adjustment for dietary factors had minor impact on the associations (OR per 1 daily serving of processed meat was 1.26, 95% CI 1.06–1.51 for LADA; and 1.09, 95% CI 0.94–1.25, for T2D). Neither did mutual adjustment for unprocessed and processed meat alter the observed associations between red meat intake and LADA or T2D. The increased risk of LADA per one daily serving increment in processed meat intake was observed also when restricting the analysis to patients responding to the questionnaire within 6 months of diabetes diagnosis (OR 1.21, 95% CI 0.95–1.55). Similarly, the results did not change materially neither when excluding patients reporting ‘diet modification’ as current diabetes treatment (OR per additional serving was 1.21, 95% CI 0.99–1.47, for LADA), nor when excluding patients reporting ‘major lifestyle changes’ after diagnosis (LADA *n* = 156, OR 1.24, 95% CI 1.01–1.52).

## Discussion

These novel findings indicate that consumption of processed red meat increases the risk of LADA, especially in combination with high-risk HLA genotypes or FHD-T1D. In contrast, for T2D no associations with processed or unprocessed red meat were observed, and neither *TCF7L2* genotype nor FHD-T2D influenced these findings. Processed red meat consumption was, however, associated with increased insulin resistance and worse beta cell function among individuals with T2D, with similar but non-significant associations for LADA.

Our findings are in line with previous observations in T1D in children; Recently, Syrjälä and colleagues demonstrated a positive association between total meat intake and islet autoimmunity and childhood T1D [4]. Furthermore, in a case–control study, an increased risk of T1D was found in relation to total meat intake [2]. Maternal intake of red meat and meat products during lactation [3], but not during pregnancy [24–26], has been positively associated with islet autoimmunity or/and T1D. Moreover, biomarkers of red meat fat [7], but not the meat intake [27] were associated with autoimmunity in genetically susceptible children.

An increased risk of LADA observed for intake of processed, but not unprocessed, red meat may suggest that underlying mechanisms are related to compounds specific to, or found at higher levels in, processed meat products. Furthermore, the highest risk of LADA in relation to processed red meat intake was observed when combined with high-risk HLA genotype or FHD-T1D. Indeed, many of the compounds hypothesized to be responsible for a positive association with both autoimmune diabetes and T2D are more abundant in processed red meat with suggested mechanisms involving adverse effects on beta cell function. This may be a potential explanation for our finding; individuals at high genetic risk may already have compromised beta cell function and hence be more susceptible to the adverse effects of high processed meat intake. The lack of interaction between processed red meat intake and genotypes of *TCF7L2* or FHD-T2D on the risk of LADA, and the overall lack of association regarding T2D, support the potential involvement of autoimmune-related genetic susceptibility. Compounds that potentially link red meat intake to diabetes risk include AGEs, which are proinflammatory compounds formed in the preparation and cooking processes of red meat, particularly processed meat. In rodent studies, high AGE diets have been demonstrated to induce insulin secretory dysfunction, and a low AGE diet decreased the incidence of autoimmune diabetes in nonobese diabetic mice and improved insulin resistance and beta cell function in mice with T2D [28]. Other compounds of interest in processed meat products are nitrates and nitrites, which are added as preservatives and potentially converted to nitrosamines [5]. Toxic effect of nitrosamines on beta cells has been supported by studies in rodents [29] and dietary nitrites and nitrosamines have been positively associated with childhood T1D in case–control studies [30, 31]. Potential adverse effects have also been ascribed sodium, which is another micronutrient found at high levels in processed meat and has been associated with T2D risk and insulin resistance [32].

Several prospective studies have reported that consumption of red meat, particularly processed red meat, is associated with increased risk of T2D [33]. We found a similar positive association, but this was fully attenuated after adjustment for BMI. This may suggest that the association is mediated through effects on BMI. In fact, BMI has previously been suggested to partly explain the associations between red meat intake and T2D [34–36], and red meat intake has been positively associated with weight gain [37]. However, contrary to our findings, in these studies the associations remain, although attenuated, also after adiposity adjustment. One possible explanation may be that longitudinal studies often adjust for historical BMI (assessed at baseline or other prior diagnosis timepoint), whereas we adjust for BMI at diagnosis (index time for controls) which may yield more complete adjustment. The fact that we observed increased insulin resistance and worse beta cell function in relation to red meat consumption for T2D, with similar indications also for LADA, speak in favour of direct adverse effects. There were no indications that consumption of processed red meat affect LADA risk by triggering autoimmunity. In this context it is worth noting that a recent publication based on prospective data found no association between red meat consumption and risk of rheumatoid arthritis [38]. An association with insulin resistance is supported by a recent longitudinal study reporting positive associations, also after adiposity adjustment, for HOMA-IR in relation to total animal protein and protein from meat [39]. We did not observe any interaction between processed red meat intake and genotypes or FHD in relation to T2D. This contrasts a previous U.S. study which found an interaction with a T2D-GRS including *TCF7L2* (rs12255372) [6] but is in line with a Swedish study using a different GRS also including *TCF7L2* (rs7903146) [40].

Strengths of the present study include the population-based design, extensive information on diet and essential confounding factors, and the large number of incident cases of LADA. Given that LADA is relatively rare, a case–control design is an efficient way to achieve enough cases for viable analyses [14]. However, since exposure data is collected retrospectively, recall bias is a concern. Importantly, dietary data was collected in close conjunction with diagnosis and participants were instructed to report their habits preceding diagnosis. Furthermore, the observed associations remained in sensitivity analysis restricted to patients with the shortest duration of diabetes at study inclusion. In addition, our results for T2D before adjustment for BMI are in line with previous, prospective studies [34, 36]. Another concern is that patients may have made dietary modifications following diagnosis and reported their modified dietary regimen. However, this would lead to overestimations of the observed positive associations only if patients had increased their intakes of red meat after diagnosis. This seems unlikely; the national guidelines for diabetes patients recommend increased intake of unsaturated fats (at the expense of saturated fats) and the general dietary guidelines recommend limiting the intake of red meat, particularly processed meat, as part of a healthy diet. Of note, the observed ORs were largely unchanged in sensitivity analyses excluding patients reporting being on ‘diet modification’ treatments or having had major lifestyle changes after diagnosis. The use of external genetic controls and the fact that it was not possible to adjust for energy intake in the interaction analysis with HLA and *TCF7L2* is a limitation, but energy intake did not seem to have a great impact on the estimates in the main analysis using the internal controls. The genetic controls had slightly lower processed red meat intake compared to the internal controls (mean: 0.7 serv/day and 0.8 serv/day, respectively), which may potentially lead to overestimated OR associated with processed red meat intake. However, any such impact on the study findings seem limited, since the interaction analyses with genotypes showed similar results as those obtained in interaction analyses, where FHD was used as an indicator of genetic susceptibility, i.e., when the ESTRID controls were used and energy intake was accounted for.

In conclusion, we present novel findings that consumption of processed red meat may be a risk factor for LADA, independent of adiposity, and potentially most detrimental in individuals with HLA-conferred susceptibility or FHD-T1D. These findings add support for a role of processed red meat in the development of autoimmune diabetes in children and adults. This is important considering that established modifiable risk factors for primary prevention of autoimmune diabetes are still lacking.

## Electronic supplementary material

Below is the link to the electronic supplementary material.

Supplementary file1 (DOCX 286 kb)
