# Supplementary material for: Consumption of red meat, genetic susceptibility, and risk of LADA and type 2 diabetes
Source: Eur J Nutr. 2020 May 22;60(2):769–79. doi: 10.1007/s00394-020-02285-2 (PMC7900036; doi:10.1007/s00394-020-02285-2)
Supplement: Supplementary file 1 — Supplementary file1 (DOCX 286 kb) [file 394_2020_2285_MOESM1_ESM.docx]

European Journal of Nutrition

Electronic Supplementary Material for manuscript

*Consumption of red meat, genetic susceptibility, and risk of LADA and type 2 diabetes*

Löfvenborg JE, Ahlqvist E, Alfredsson L, Andersson T, Groop L, Tuomi T, Wolk A, Carlsson S

Corresponding author

Josefin Edwall Löfvenborg

Affiliation: Karolinska Institutet, Stockholm, Sweden

E-mail: josefin.lofvenborg@ki.se

**Supplementary Figure 1.** Schematic of the ESTRID case-control study design.

**
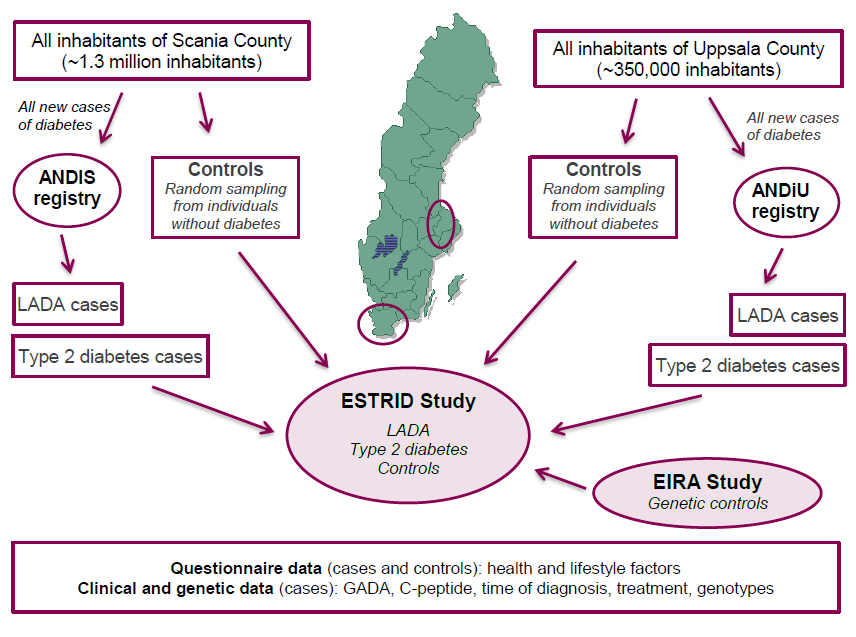
**

**Supplementary Table 1.** Characteristics of study participants (cases and internal controls) in ESTRID according to quartiles of unprocessed and processed red meat intake. All numbers are means (SD) unless otherwise stated.

|  | Unprocessed red meat | | | |  | Processed red meat | | | |
| --- | --- | --- | --- | --- | --- | --- | --- | --- | --- |
| Servings per day, quartile | 1 | 2 | 3 | 4 |  | 1 | 2 | 3 | 4 |
| n (%) | 948 | 957 | 908 | 969 |  | 852 | 905 | 940 | 1085 |
| Unprocessed red meat, serv/d | 0.21 (0.09) | 0.43 (0.05) | 0.62 (0.05) | 0.86 (0.35) |  | 0.39 (0.33) | 0.54 (0.26) | 0.58 (0.27) | 0.65 (0.38) |
| Processed red meat, serv/d | 0.56 (0.57) | 0.86 (0.63) | 0.96 (0.67) | 1.07 (0.69) |  | 0.18 (0.11) | 0.51 (0.10) | 0.87 (0.13) | 1.69 (0.61) |
| Total red meat, serv/d | 0.77 (0.60) | 1.28 (0.63) | 1.58 (0.67) | 2.01 (0.82) |  | 0.57 (0.35) | 1.05 (0.29) | 1.45 (0.30) | 2.34 (0.76) |
| Age, years | 63.6 (11.9) | 61.3 (12.6) | 59.4 (12.3) | 57.7 (11.8) |  | 60.9 (12.5) | 59.5 (12.6) | 59.6 (12.2) | 61.9 (12.1) |
| Sex, % women | 48.4 | 48.9 | 46.9 | 40.1 |  | 56.9 | 49.4 | 43.7 | 36.8 |
| High level of education, % university | 25.7 | 27.7 | 29.4 | 33.5 |  | 36.3 | 30.1 | 28.6 | 23.1 |
| BMI current, kg/m^2^ | 28.1 (5.5) | 28.0 (5.3) | 28.5 (5.6) | 28.6 (5.4) |  | 27.5 (5.4) | 28.0 (5.2) | 28.6 (5.3) | 29.0 (5.8) |
| BMI at age 20, kg/m^2^ | 21.7 (3.2) | 21.8 (3.2) | 22.1 (3.4) | 22.0 (3.3) |  | 21.5 (3.2) | 21.8 (3.6) | 22.0 (3.0) | 22.2 (3.2) |
| Av. annual weight change (%) since 20 | 0.74 (0.66) | 0.78 (0.66) | 0.79 (0.70) | 0.86 (0.73) |  | 0.75 (0.71) | 0.78 (0.69) | 0.82 (0.68) | 0.81 (0.68) |
| Physically inactive, % | 19.3 | 18.9 | 17.3 | 19.8 |  | 19.8 | 16.4 | 19.0 | 20.0 |
| Family history of diabetes, % yes | 36.4 | 38.0 | 34.6 | 38.2 |  | 36.5 | 36.6 | 35.3 | 38.6 |
| Smoking status, % current | 17.9 | 19.8 | 19.8 | 22.5 |  | 22.2 | 20.2 | 18.4 | 19.5 |
| Energy intake among women, kcal/d | 1439 (455) | 1570 (433) | 1625 (444) | 1821 (564) |  | 1418 (445) | 1577 (463) | 1644 (458) | 1825 (516) |
| Energy intake among men, kcal/d | 1702 (540) | 1964 (574) | 2206 (623) | 2344 (688) |  | 1673 (547) | 1905 (562) | 2145 (623) | 2324 (675) |
| Alcohol, g/d | 7.3 (8.6) | 7.9 (9.5) | 8.8 (9.3) | 10.2 (11.0) |  | 6.5 ( 8.4) | 8.6 (9.6) | 9.1 (10.0) | 9.6 (10.3) |
| Coffee, serv/d | 2.83 (2.21) | 3.04 (2.13) | 3.22 (2.18) | 3.30 (2.43) |  | 2.74 (2.18) | 3.00 (2.14) | 3.21 (2.22) | 3.35 (2.38) |
| Sweetened beverages, serv/d | 0.33 (0.91) | 0.44 (1.18) | 0.52 (1.43) | 0.63 (1.34) |  | 0.35 (1.04) | 0.46 (1.11) | 0.51 (1.24) | 0.57 (1.45) |
| Sugar-sweetened beverages, serv/d | 0.15 (0.49) | 0.23 (0.91) | 0.30 (1.25) | 0.34 (1.00) |  | 0.16 (0.73) | 0.22 (0.70) | 0.29 (0.98) | 0.33 (1.22) |
| Vegetables, serv/d | 3.12 (2.31) | 3.18 (1.91) | 3.44 (1.85) | 3.75 (2.33) |  | 3.36 (2.43) | 3.28 (1.98) | 3.31 (1.92) | 3.51 (2.16) |
| Fruits, serv/d | 1.45 (1.23) | 1.44 (1.12) | 1.49 (1.22) | 1.46 (1.19) |  | 1.49 (1.25) | 1.44 (1.29) | 1.41 (1.14) | 1.49 (1.11) |
| Fish, serv/d | 0.40 (0.34) | 0.44 (0.28) | 0.46 (0.28) | 0.53 (0.50) |  | 0.43 (0.42) | 0.44 (0.30) | 0.46 (0.29) | 0.50 (0.43) |
| Fatty fish, serv/d | 0.22 (0.23) | 0.23 (0.19) | 0.24 (0.19) | 0.28 (0.31) |  | 0.23 (0.28) | 0.23 (0.21) | 0.24 (0.18) | 0.27 (0.27) |
| Biscuits, sweets, salty snacks, serv/d | 0.87 (0.77) | 0.99 (0.79) | 1.04 (0.73) | 1.13 (0.93) |  | 0.84 (0.79) | 0.91 (0.66) | 1.03 (0.81) | 1.19 (0.91) |
| Whole grain, g/d | 49.4 (30.1) | 53.9 (30.7) | 53.8 (31.5) | 53.8 (36.5) |  | 50.2 (34.3) | 51.1 (31.4) | 52.4 (29.4) | 55.5 (33.8) |
| Total iron, mg/d | 8.6 (3.2) | 9.9 (3.2) | 11.0 (3.5) | 12.5 (4.3) |  | 8.6 (3.4) | 9.8 (3.2) | 10.8 (3.4) | 12.4 (4.1) |
| Total sodium, mg/d | 2190.2 (723.6) | 2591.0 (742.4) | 2879.3 (874.7) | 3271.3 (1043.0) |  | 2117.7 (711.6) | 2510.3 (721.2) | 2856.1 (830.1) | 3299.0 (1003.0) |
| PUFA/SFA ratio | 0.42 (0.19) | 0.39 (0.14) | 0.39 (0.13) | 0.39 (0.13) |  | 0.43 (0.20) | 0.39 (0.14) | 0.39 (0.13) | 0.39 (0.12) |
| E% fat | 32.9 (5.8) | 33.7 (5.3) | 33.8 (5.2) | 34.8 (5.6) |  | 32.3 (6.1) | 33.2 (5.1) | 34.0 (5.2) | 35.3 (5.3) |
| E% protein | 17.0 (2.9) | 17.4 (2.5) | 17.8 (2.7) | 18.3 (3.0) |  | 17.6 (3.3) | 17.8 (2.7) | 17.7 (2.7) | 17.5 (2.6) |
| E% carbohydrates | 44.0 (6.7) | 43.1 (6.1) | 42.6 (6.3) | 41.0 (6.9) |  | 44.1 (7.2) | 42.9 (6.4) | 42.4 (6.2) | 41.6 (6.3) |

**Supplementary Figure 2.** OR (95% CI) of LADA and type 2 diabetes in relation to HLA genotype, *TCF7L2* genotype, family history of type 1 diabetes, and family history of type 2 diabetes. The genetic analyses are adjusted for age and sex. Family history analyses are adjusted for age, sex, education, smoking, physical activity, alcohol intake, BMI, plus mutual adjustment for type 1 and type 2 diabetes.


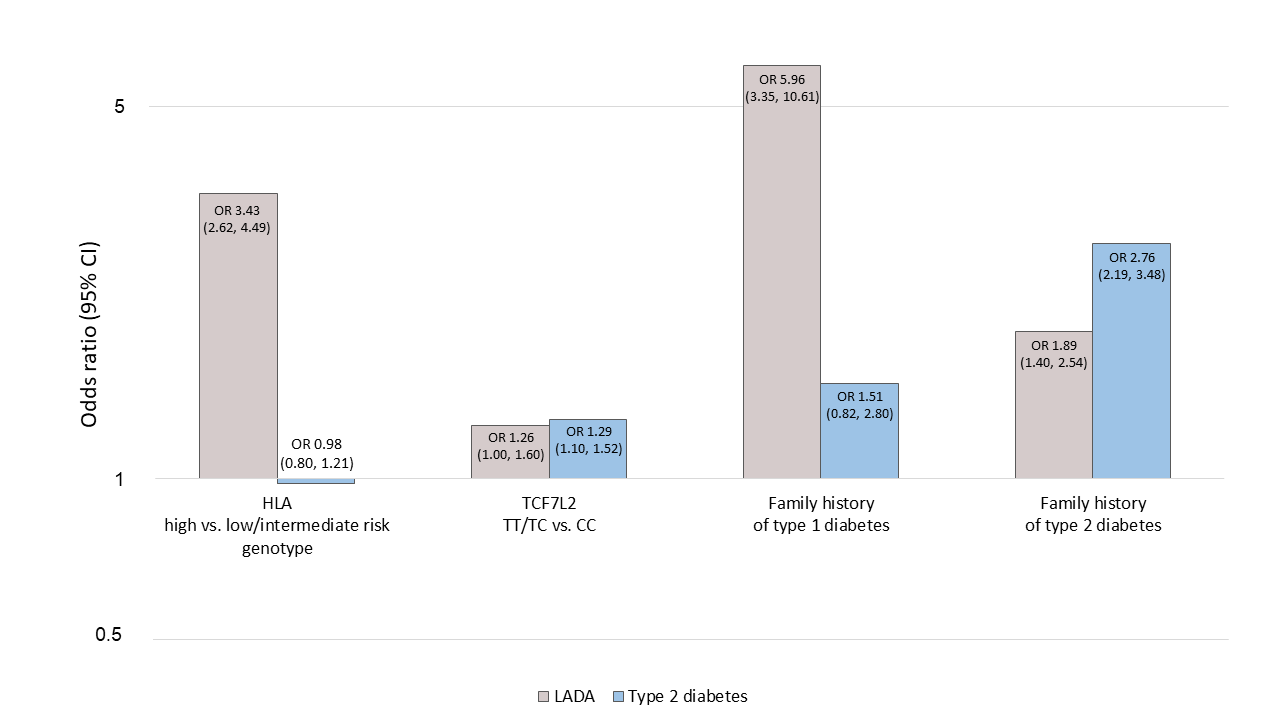


**Supplementary Table 2.** Change (as percentage) in HOMA-B, HOMA-IR, and GADA per one serving/day increment in unprocessed and processed red meat intake. Analyses were adjusted for age, sex, education, smoking, physical activity, alcohol, energy intake, family history of diabetes, and BMI.

|  | **LADA** | | | | | |  | **Type 2 diabetes** | | | |
| --- | --- | --- | --- | --- | --- | --- | --- | --- | --- | --- | --- |
|  | HOMA-B | | HOMA-IR | | GADA | |  | HOMA-B | | HOMA-IR | |
| Per 1 serving/day increment | % change | *p* | % change | *p* | % change | *p* |  | % change | *p* | % change | *p* |
|  |  |  |  |  |  |  |  |  |  |  |  |
| Unprocessed red meat | -8.7 | 0.6148 | 6.9 | 0.7119 | 82.6 | 0.1578 |  | -12.2 | 0.0096 | -2.5 | 0.6259 |
|  |  |  |  |  |  |  |  |  |  |  |  |
| Processed red meat | -9.6 | 0.1257 | 11.5 | 0.0980 | -0.5 | 0.9733 |  | -6.7 | 0.0056 | 8.1 | 0.0029 |
|  |  |  |  |  |  |  |  |  |  |  |  |
